# Anti-Inflammatory Effect of Sulforaphane on LPS-Activated Microglia Potentially through JNK/AP-1/NF-κB Inhibition and Nrf2/HO-1 Activation

**DOI:** 10.3390/cells8020194

**Published:** 2019-02-22

**Authors:** Lalita Subedi, Jae Hyuk Lee, Silvia Yumnam, Eunhee Ji, Sun Yeou Kim

**Affiliations:** 1 Laboratory of Pharmacognosy, College of Pharmacy, Gachon University, #191, Hambakmoero, Yeonsu-gu, Incheon 21936, Korea; subedilali@gmail.com (L.S.); wogur6378@naver.com (J.H.L.); silviayumnam@gmail.com (S.Y.); 2 Laboratory of Clinical Pharmacy, College of Pharmacy, Gachon University, #191, Hambakmoero, Yeonsu-gu, Incheon 21936, Korea; ehji@gachon.ac.kr; 3 Gachon Institute of Pharmaceutical Science, Gachon University, 191, Hambakmoe-ro, Yeonsu-gu, Incheon 21936, Korea; 4 Gachon Medical Research Institute, Gil Medical Center, Incheon 21565, Korea

**Keywords:** neuroinflammation, microglial activation, sulforaphane, pre-treatment, post-treatment

## Abstract

Sulforaphane (SFN), a potent nuclear factor erythroid 2-related factor 2 (Nrf2) activator, is present in the species of the Brassicaceae, especially in broccoli sprouts. In this study, the effects of SFN against microglial activation and inflammation, and the potential mechanisms involved, were analyzed. As mitogen-activated protein kinase (MAPK) signaling plays a key role in microglial activation and inflammation, we focused on the role of SFN in regulating the MAPK signaling regulation of the inflammatory and anti-inflammatory cascades in lipopolysaccharide (LPS)-activated microglia. The anti-inflammatory and immunomodulatory effects of SFN were explored by evaluating the expression and secretion of inflammatory proteins, cytokines, nuclear factor kappa-light-chain-enhancer of activated B cells (NF-κB), and activator protein-1 (AP-1) under pre- and post-treatment conditions. Under the SFN pre- and post-treatment conditions, the MAPK phosphorylation levels were significantly reduced in both acutely and chronically activated microglial cells. SFN also reduced the c-Jun N-terminal kinase (JNK) phosphorylation levels, which subsequently reduced NF-κB and AP-1 signaling. As a result, the expression of the inflammatory mediators (iNOS, COX-2, NO, and PGE2) and proinflammatory cytokines (TNF-α, IL-6, and IL-1β) was decreased. At the same time, SFN increased the expression of Nrf2 and heme oxygenase-1 (HO-1) as well as the production of the anti-inflammatory cytokines IL-10 and IL-4. In conclusion, this study demonstrated that SFN exerts an anti-neuroinflammatory effect on microglia through JNK/AP-1/NF-κB pathway inhibition and Nrf2/HO-1 pathway activation.

## 1. Introduction

Sulforaphane (SFN), an active phytochemical and nutraceutical present in members of the Brassicaceae family (e.g., broccoli and cabbage), is a product of the catalytic conversion of glucoraphanin by the enzyme myrosinase [1]. Aside from being a well-known nuclear factor erythroid 2-related factor 2 (Nrf2) activator, SFN has also shown various biological activities, including anti-inflammatory and anticancer effects [2]. The rich presence of SFN in broccoli allows for its ready bioavailability to individuals suffering from neurodegenerative diseases who could benefit from the potential neuroprotective effects of this phytochemical [3]. SFN shows a biological potency through various pathways in both a Nrf2-dependent and -independent manner. Greaney et al. reported that SFN inhibited multiple inflammasomes through Nrf2-independent pathways [4]. Eren et al. suggested that SFN induced anti-inflammatory effects against lipopolysaccharide (LPS)-activated inflammation via extracellular signal-regulated kinases (ERK)/Nrf2 activation and tumor necrosis factor-alpha (TNF-α) inhibition [5]. SFN inhibited the inflammation and proliferation of smooth muscle cells through the inhibition of TNF-α and nuclear factor kappa-light-chain-enhancer of activated B cells (NF-κB) signaling [6]. Mitogen-activated protein kinases (MAPK), such as p38, c-Jun NH_2_-terminal kinases (JNK), and ERK, induce the production of inflammatory cytokines, especially TNF-α [7]. More specifically, JNK signaling is involved in a cascade wherein TNF-α induction and upregulated inflammatory mediators cause neuroinflammation/neurodegeneration [8]. Neuronal degeneration can be caused by JNK phosphorylation, followed by the activator protein 1 (AP-1)- and NF-κB-mediated transcription of inflammatory mediators, such as nitric oxide (NO), inducible nitric oxide synthase (iNOS), cyclooxygenase-2 (COX-2), and prostaglandin E2 (PGE2), and the proinflammatory cytokines TNF-α, interleukin (IL)-6, and IL-1β. This neurodegeneration is characteristic of disorders, such as Alzheimer’s disease and Parkinson’s disease, demyelination in multiple sclerosis, and M1 microglial polarization in ischemia. More specifically, the JNK pathway is considered as a hallmark of neuronal cell death. Alteration of JNK phosphorylation consequently inhibits the c-Jun-mediated AP-1 transcription of proapoptotic proteins of the Bcl2 family [9]. Dhanasekaran and Reddy also suggested that JNK activation is responsible for the activation of the extrinsic and intrinsic apoptotic pathways that are responsible for apoptotic signaling [10]. Previously, we also observed that LPS-treated microglia significantly activated the MAPK signaling that was responsible for significant neuronal death [11]. Similarly, our previous findings revealed that *N*,*N*-disubstituted azine inhibited JNK phosphorylation, which was followed by the significant inhibition of TNF-α production as well as of the neuronal necrosis and apoptosis mediated through activated microglia-induced toxicity [12]. Previous literature has already pointed out that SFN-enriched broccoli and SFN itself have the potential to inhibit neuronal death and cell damage [13,14,15]. In addition to this, the activation of Nrf2 and heme oxygenase-1 (HO-1) is known to potentiate the production of anti-inflammatory cytokines, particularly IL-10, thereby indicating the anti-inflammatory potential of SFN [16,17]. The aim of this study was therefore to elucidate the exact mechanism behind the anti-inflammatory activity of SFN. With these prior studies as a backdrop, we evaluated the roles of SFN against LPS-induced microglial activation and in regulating MAPK signaling, AP-1- and NF-κB-mediated transcription, and the expression of the inflammatory mediators iNOS, COX-2, PGE2, NO, TNF-α, IL-6, and IL-1β. 

According to previous reports, a potent JNK inhibitor may inhibit not only inflammation but also neurotoxicity. In this study, we provide evidence to prove that SFN modulates inflammatory responses by inhibiting the JNK, AP-1, and NF-κB pathways in LPS-activated BV2 microglia.

## 2. Materials and Methods

### 2.1. Reagents

Dulbecco’s modified Eagle’s medium (DMEM), supplemented with fetal bovine serum (FBS) and penicillin–streptomycin, was provided by Invitrogen (Carlsbad, CA, USA). LPS, allyl isothiocyanate (AITC), and SFN were obtained from the Sigma Chemical Company (St. Louis, MO, USA). Enzyme-linked immunosorbent assay (ELISA) kits for measuring IL-6, PGE2, TNF-α, and IL-1β were acquired from R&D Systems (Minneapolis, MN, USA). Primary antibodies for iNOS were acquired from Abcam (Cambridge, UK). Antibodies for COX-2, β-actin, histone-H3, c-Jun, phosphorylated c-Jun (pc-Jun), c-Fos, and phosphorylated c-Fos (pc-Fos) were acquired from Santa Cruz Biotechnology (Dallas, TX, USA). Antibodies for α-tubulin, JNK, p38, ERK, IκB, p-IκB, and NF-κB were purchased from Cell Signaling Technology (Beverly, MA, USA). The 3-(4,5-Dimethylthiazol-2-yl)-2,5-diphenyltetrazolium bromide (MTT) was purchased from Sigma-Aldrich (St. Louis, MO, USA), the radioimmunoprecipitation assay (RIPA) buffer from iNtRON Biotechnology (Seongnam-si, Gyeonggi-do, Korea), the Roswell Park Memorial Institute (RPMI) 1640 medium from MOREBIO (Seongnam-si, Gyeonggi-do, Korea), and the *N*-monomethyl-l-arginine (l-NMMA) from the Sigma Chemical Company (St. Louis, MO, USA).

### 2.2. Cell Culture

BV2 cells, used as representative microglial cells, were kindly provided by Dr. E. Choi at Korea University (Seoul, Korea). The cells were maintained in a high-glucose DMEM supplemented with 10% FBS and 1% of an antibiotic mix (1 × 10^5^ units/L penicillin and 100 mg/L streptomycin), at 37 °C in a humidified incubator with 5% CO_2_ for their proper growth and stability. The RAW264.7 cells were purchased from the American Type Culture Collection (ATCC) (Manassas, VA, USA), whereas the THP-1 cells were purchased from the Korea Cell Line Bank (Seoul, Korea). 

### 2.3. Cell Treatment and Nitrite and Cell Viability Assays

MTT assays were conducted to evaluate the cytotoxicity of the treated compounds [18]. Cells were seeded in 96-well plates and incubated overnight, following which the seeded cells were treated for 30 min with different concentrations of SFN or AITC, before or after LPS (100 ng/mL) activation. Normal cells that were not treated with the compounds and not activated with LPS were used as the control group. The cells were incubated for another 24 h or as required, according to experimental planning. On the next day, 50 µL of conditioned medium (CM) from each well was transferred to a new 96-well plate and mixed with an equal volume of Griess reagent (1% sulfanilamide and 0.1% *N*-(1-naphthyl) ethylenediamine dihydrochloride) for the measurement of nitrite production. Sodium nitrite was used as a positive control and the change in color was measured as the optical density (OD) at 540 nm in a microplate reader. The adherent cells in the treated plate were incubated with MTT (0.5 mg/mL) for almost 1 h, following which the solution was removed and the cells, stained blue, were exposed to 200 µL of dimethyl sulfoxide, resulting in the formation of the purple-colored formazan. This color change was used as a measurement of cell viability by measuring the OD at 570 nm using a microplate reader. The results were expressed as a percentage of the LPS-treated cells (i.e., the LPS-treated group as 100%).

### 2.4. Western Blot Analysis

**Scheme 1:**
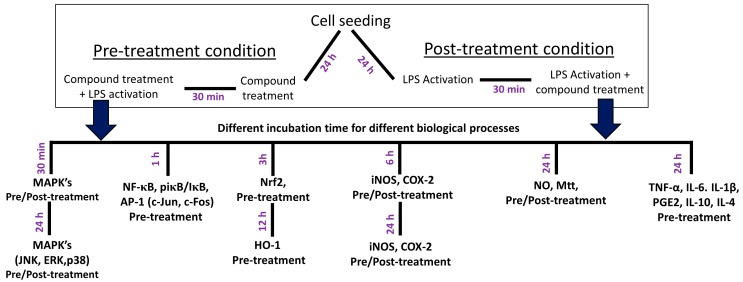
Scheme of experimental procedure. Microglial cells were treated with a tested compound followed by LPS activation for pre-treatment, while LPS activation was followed by a compound treatment for the post-treatment condition. The time point for the determination of different biomarkers was varied. MAPKs were evaluated after 30 min and 24 h, whereas iNOS and COX-2 were evaluated at 6 h and 24 h after LPS exposure. NF-kB and AP-1 related proteins were evaluated after 1 h of incubation, while Nrf2 and HO-1 were evaluated at 3 h and 12 h after incubation, respectively. The secreted level of NO, PGE2, TNF-α, IL-6, IL-1β, IL-4, and IL-10 was evaluated in the conditioned medium 24 h after LPS exposure.

The expression of inflammatory proteins was analyzed using the western blot assay. Cells were seeded into plates, treated, and incubated to the desired time point. The treatment times for the different biological processes are shown in Scheme 1. The treated cells were then washed, collected, and lysed with the RIPA lysis buffer containing protease and phosphatase inhibitors. Protein estimation was performed using the Bradford assay, and 30 μg of protein from each sample was separated by sodium dodecyl sulfate-polyacrylamide gel electrophoresis (SDS-PAGE). The protein bands in the gel were transferred to nitrocellulose membranes and incubated overnight with primary antibodies against α-tubulin, iNOS, COX-2, ERK, pERK, JNK, pJNK, p38, p-p38, NF-κB, histone-H3, β-actin, IκB, pIκB, c-Fos, pc-Fos, c-Jun, or pc-Jun. On the next day, the membranes were washed and then incubated with the respective secondary antibodies, and the protein bands were finally visualized via the Chemiluminesent ECL Western Blotting Detection Reagent (Amersham Pharmacia Biotech, Little Chalfont, UK). A quantitative evaluation of the bands was conducted using the Image Master™ 2D Elite software (version 3.1, Amersham Pharmacia Biotech, Little Chalfont, UK).

### 2.5. Measurement of PGE2, TNF-α, IL-1β, IL-6, IL-10, and IL-4 Production

CM from the treated cells was used to evaluate the levels of secreted inflammatory mediators and proinflammatory cytokines, such as PGE2, TNF-α, IL-1β, and IL-6. For this purpose, BV2 cells were seeded in a 6-well or a 24-well plate and treated, as described previously. The treated and LPS-activated cells were incubated for 24 h, whereupon the CM was collected. The cell viability assay was performed on the attached cells, whereas competitive ELISA was used to measure the levels of PGE2, TNF-α, IL-1β, and IL-6 secreted into the CM. ELISA development kits (R&D Systems) were used to measure TNF-α, IL-1β, and IL-6 secretion, whereas a competitive enzyme immunoassay kit (Cayman Chemical, Ann Arbor, MI, USA) was used to measure PGE2, according to the manufacturers’ instructions. IL-10 and IL-4 secretion was measured using the Mouse Th1/Th2 Uncoated ELISA Kit (Invitrogen).

### 2.6. NF-κB Assay

The translocation of NF-κB and expressions of IκB and pIκB in the microglial cells were evaluated after 1 h of LPS activation. The cells were harvested, and the cytosolic and nuclear extracts were collected using the Nuclear/Cytosolic Extraction Kit (Active Motif, Carlsbad, CA, USA), according to the manufacturer’s instructions. The nuclear and cytosolic proteins (30 µg) were separated using SDS-PAGE, and the protein expression levels of NF-κB, histone-H3, IκB, and pIκB were evaluated. Histone-H3 was used as a loading control for the nuclear proteins, whereas Glyceraldehyde 3-phosphate dehydrogenase GAPDH was used as a loading control for the cytosolic proteins. Densitometric analyses of the bands were performed using the Image Master™ 2D Elite software (version 3.1).

### 2.7. Statistical Analysis

All data are presented as the mean ± standard error of the mean. The statistical significance of differences between the groups was determined by a one-way analysis of variance, followed by the Tukey post hoc test, using GraphPad Prism 5 (GraphPad Software Inc., La Jolla, CA, USA), and was set at *p* < 0.05. 

## 3. Results

### 3.1. SFN Inhibited Nitrite Production and iNOS and COX-2 Expression in LPS-Activated Microglial Cells

**Figure 1:**
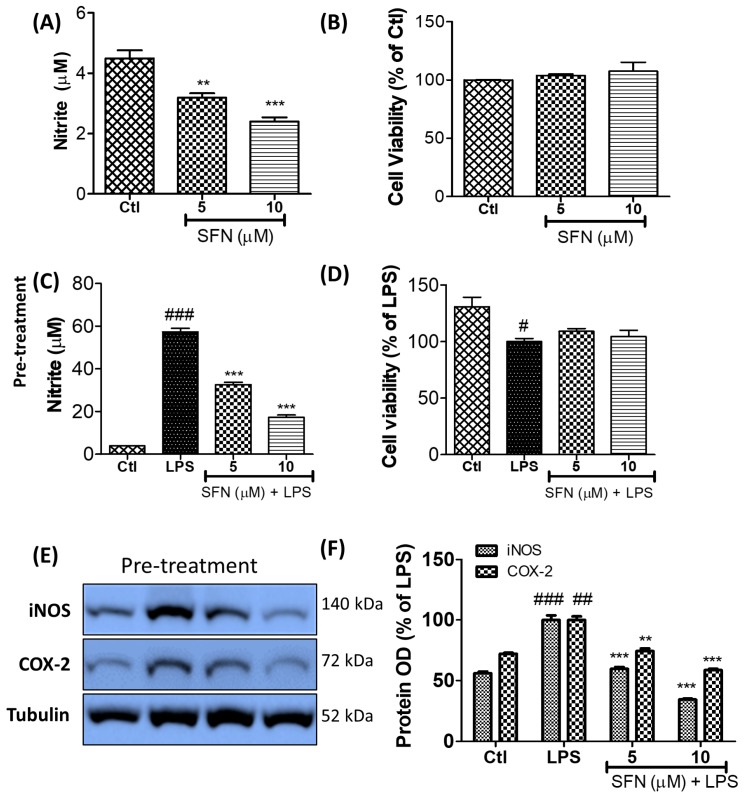
SFN inhibited nitrite production and iNOS and COX-2 expression in LPS-activated murine microglial cells without cellular toxicity. (**A**,**B**) Nitrite concentrations and percentage cell viability of normal microglia after treatment with two different concentrations of SFN alone. (**C**,**D**) Nitrite concentrations and percentage cell viability of LPS-activated BV2 microglial cells. The cells were pre-treated with SFN and then activated with LPS (100 ng/mL) for 6 h. (**E**,**F**) Quantification of iNOS and COX-2 expression in the LPS-activated BV2 cells. All data are presented as the mean ± standard error of the mean of three independent experiments. ** *p* < 0.01 and *** *p* < 0.001 indicate significant differences compared with LPS treatment alone; # *p* < 0.05, ## *p* < 0.01, and ### *p* < 0.001 indicate significant differences compared with the untreated control group. Ctl—untreated control cells; LPS—cells treated with lipopolysaccharide only.

LPS treatment activates the microglia, resulting in the production of NO, the increased accumulation of which is a key biomarker for inflammation. Such increased NO production necessitates an increase in iNOS and is directly responsible for the activation of COX-2. As shown in Figure 1, LPS treatment significantly increased the expression of iNOS and COX-2 in the BV2 microglial cells, but this effect was reversed by the SFN treatment. The phytochemical also inhibited nitrite (and hence NO) production with an IC_50_ value of 5.85 µM. As the immune cell lines RAW264.7 and THP-1 also respond to LPS for activating and inducing inflammation, the inhibitory effect of SFN on NO production and iNOS and COX-2 expression was also confirmed in these cells (Supplementary Figure S1). SFN inhibited NO production in the RAW 264.7 and THP-1 cells with IC_50_ values of 7.14 and 6.76 µM, respectively. In addition to this, SFN significantly inhibited the expression of iNOS and COX-2, suggesting that this phytochemical could potentially mediate anti-inflammatory activity in LPS-activated myeloid-derived cell lines. Several other observations supported the measured NO inhibition after SFN treatment. The SFN-mediated inhibition of iNOS was higher than that of COX-2 in all the cells. This significant inhibition of iNOS and COX-2 in all the treated cells, followed by NO inhibition, revealed the immunomodulatory potency of SFN in immune cells, such as microglia, macrophages, and monocytes. As these results suggested the possibility that SFN has a great potency to downregulate neuroinflammation, our further experiments focused on LPS-activated microglia.

### 3.2. SFN Inhibited Nitrite Production in LPS-Activated Microglial Cells

**Figure 2:**
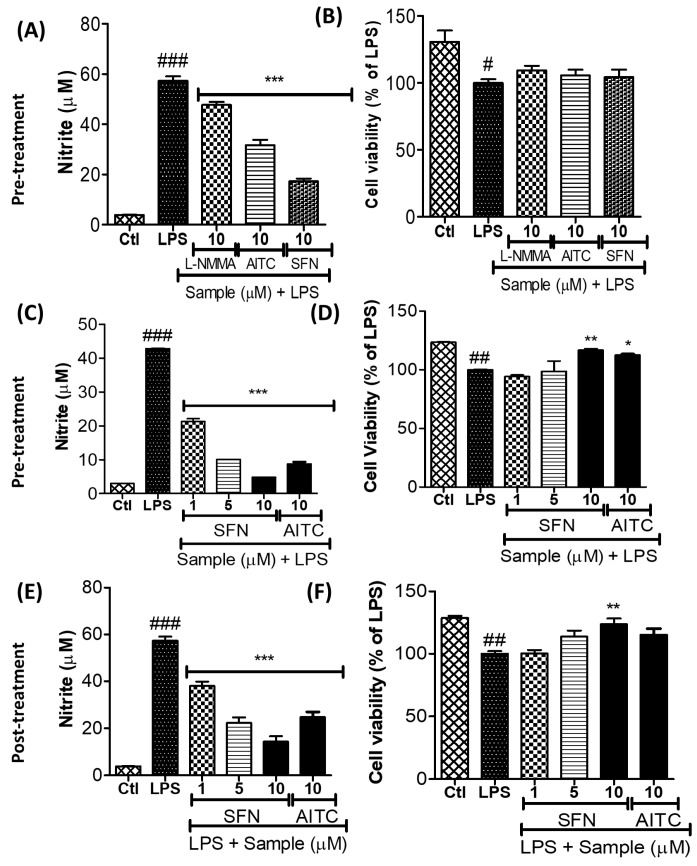
SFN inhibited nitrite production in LPS-activated BV2 microglial cells without cellular toxicity. BV2 cells were pre-/post-treated with different chemicals followed by the LPS (100 ng/mL) activation for a total of 24 h. (**A**,**B**) Comparison of nitrite inhibition by SFN, AITC, and l-NMMA pre-treatment (Sample (µM) + LPS) in LPS-activated microglia. (**C**,**D**) Nitrite concentrations and percentage cell viability after SFN or AITC pre-treatment. (**E**,**F**) Nitrite concentrations and percentage cell viability after SFN or AITC post-treatment. All data are presented as the mean ± standard error of the mean of three independent experiments. * *p* < 0.05, ** *p* < 0.01, and *** *p* < 0.001 indicate significant differences compared with LPS treatment alone; # *p* < 0.05, ## *p* < 0.01, and ### *p* < 0.001 indicate significant differences compared with the untreated control group. Ctl—untreated control cells; LPS—cells treated with lipopolysaccharide only.

As indicated in Section 3.1, SFN inhibited NO production in the LPS-activated microglia without cellular toxicity. Since l-NMMA is an iNOS inhibitor, we compared its effect with that of SFN, which is a well-known nitrite inhibitor. SFN was almost 4-fold more potent than the l-NMMA positive control. Since AITC is also present in *Brassica* plants, its effect was also evaluated. SFN was almost 2-fold more potent than AITC in inhibiting nitrite production in the LPS-activated microglia. Once we had confirmed the highest potency of SFN, we compared its effects with those of AITC against LPS-mediated neuroinflammation during pre-treatment (i.e., as a prophylactic strategy) and post-treatment (i.e., as a therapeutic strategy). LPS activation was performed 30 min after the compound treatment for the pre-treatment condition, whereas it was conducted 30 min before the compound treatment for the post-treatment condition. SFN and AITC respectively showed a significant potency in inhibiting NO production, under both treatment conditions. In addition to this, the respective AITC and SFN treatments significantly increased the viability of BV2 cells that had been subjected to LPS-induced toxicity (Figure 2). 

### 3.3. SFN Significantly Inhibited iNOS and COX-2 Expression, in 6 h and 24 h of LPS Activation, under Both Prophylactic and Therapeutic Strategies

**Figure 3:**
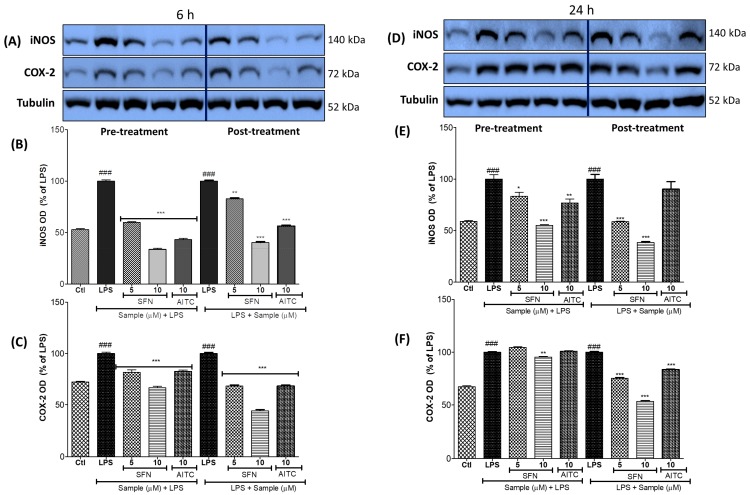
SFN inhibited the expression of iNOS and COX-2 in LPS-activated BV2 microglial cells. The cells were pre-/post-treated with SFN or AITC. LPS (100 ng/mL) activation was performed for 6 or 24 h and the expression of the proteins was evaluated by western blot analysis. (**A**–**C**) Quantification of iNOS and COX-2 expression after 6 h of LPS activation. (**D**–**F**) Quantification of iNOS and COX-2 expression after 24 h of LPS activation. α-Tubulin was used as a loading control. All data are presented as the mean ± standard error of the mean of three independent experiments. * *p* < 0.05, ** *p* < 0.01, and *** *p* < 0.001 indicate significant differences compared with LPS treatment alone; ### *p* < 0.001 indicates significant differences compared with the untreated control group. Ctl— untreated control cells; LPS—cells treated with lipopolysaccharide only.

The NO production in LPS-activated microglia has been shown to be mediated by iNOS expression. Similarly, COX-2 expression controls the production of PGE2. SFN and AITC, during both pre- and post-treatment conditions at concentrations of 5–10 µM and 10 µM, respectively, significantly inhibited the expression of iNOS and COX-2 in the LPS-activated BV2 cells (Figure 3). SFN had a greater effect than AITC for the inhibition of iNOS and COX-2 expressions in pre-/post-treatment conditions. In the case of the 24 h LPS-activated cells, whereas similar patterns in iNOS expression were observed under both treatment conditions, SFN and AITC did not significantly alter COX-2 expression during the pre-treatment condition (Figure 3). Conversely, SFN significantly inhibited COX-2 production during the post-treatment of chronic (i.e., 24 h) LPS activation.

### 3.4. SFN Pre- and Post-Treatments Significantly Modulated the MAPK Signaling Pathway, Particularly pJNK, in LPS-Activated Microglial Cells

**Figure 4:**
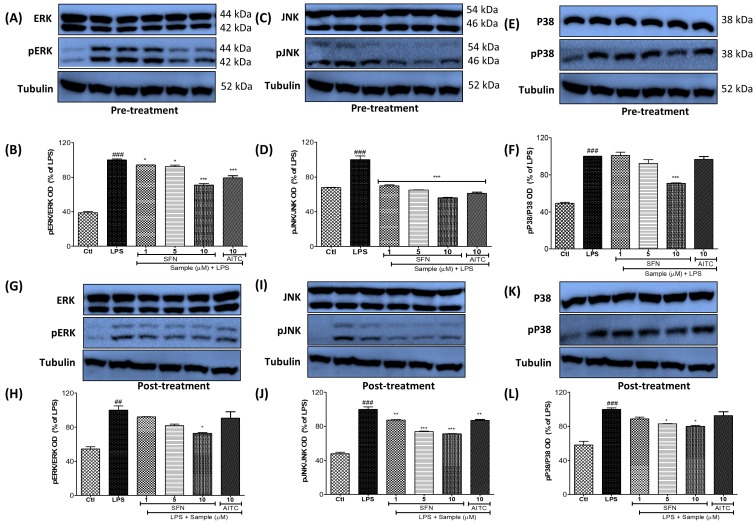
SFN modulated MAPK effector signaling after 30 min of pre-treatment (Sample (µM) + LPS) or post-treatment (LPS + Sample (µM)) in BV2 microglial cells. The cells were activated with LPS (100 ng/mL) at 30 min before/after the chemical treatments. Chemical samples of different concentrations were added in each condition. The LPS incubation was performed for 30 min. (**A**–**F**) Protein expression and band intensity of pERK/ERK, pJNK/JNK, and p-p38/p38 in LPS-activated microglia. α-Tubulin was used as a loading control. All data are presented as the mean ± standard error of the mean of three independent experiments. * *p* < 0.05, ** *p* < 0.01, and *** *p* < 0.001 indicate significant differences compared with LPS treatment alone; ## *p* < 0.01 and ### *p* < 0.001 indicate significant differences compared with the untreated control group. Ctl—untreated control cells; LPS—cells treated with lipopolysaccharide only.

Short-term (10–30 min) LPS treatment is sufficient to initiate the inflammatory cascades driven by MAPK effector signaling, activating the microglia. ERK, JNK, and p38 are target proteins of MAPK signaling, and their activation or phosphorylation is responsible for the induction of neuroinflammation. The SFN treatment (at concentrations of 1, 5, and 10 µM) nonspecifically altered the phosphorylation of JNK, ERK, and p38. However, whereas the AITC (10 µM) treatment significantly inhibited JNK phosphorylation, its inhibition of ERK and p38 phosphorylation was not significant. Consistent with the other experiments, the SFN post-treatment condition had a higher potency in inhibiting MAPK phosphorylation than the pre-treatment condition (Figure 4). The SFN-mediated inhibition of JNK was more promising in both treatment cases, suggesting that the anti-inflammatory effects of SFN might downregulate the JNK signaling pathway.

### 3.5. SFN Pre- and Post-Treatments Significantly Modulated the MAPK Signaling Pathway, Particularly pJNK and p38, in 24 h LPS-Activated Microglial Cells

**Figure 5:**
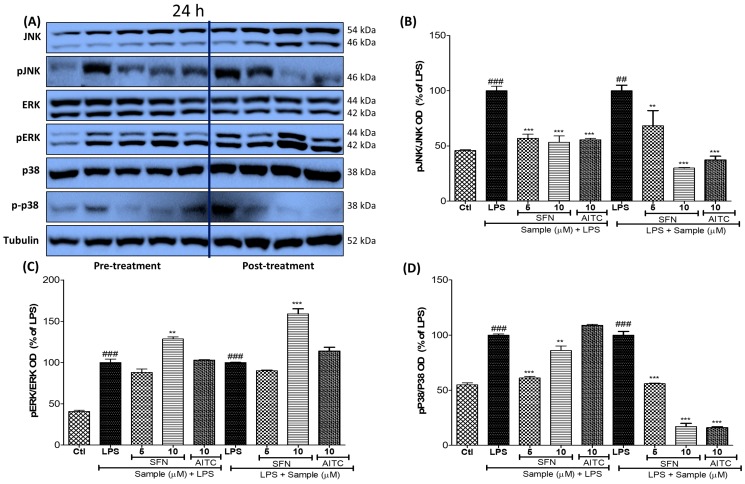
SFN pre-treatment and post-treatment modulated MAPK effector signaling in BV2 microglial cells subjected to chronic LPS (100 ng/mL) activation. The cells were pre-/post-treated with SFN or AITC, and MAPK modulation was observed after 24 h of LPS activation. (**A**–**D**) Protein expression of pJNK/JNK, pERK/ERK, and p-p38/p38. α-Tubulin was used as a loading control. All data are presented as the mean ± standard error of the mean of three independent experiments. ** *p* < 0.01 and *** *p* < 0.001 indicate significant differences compared with LPS treatment alone; ## *p* < 0.01 and ### *p* < 0.001 indicate significant differences compared with the untreated control group. Ctl—untreated control cells; LPS—cells treated with lipopolysaccharide only.

After confirming the effects of SFN on the control of MAPK effector signaling during both pre- and post-treatment conditions under acute (i.e., 6 h) LPS activation, we investigated its effects during the same conditions under chronic inflammation by activating the microglia with LPS for 24 h. Whereas its effects on JNK and p38 phosphorylation were similar, SFN treatment at 10 µM significantly increased ERK phosphorylation during chronic LPS activation under both pre- and post-treatment conditions (Figure 5). Increased ERK phosphorylation might be responsible for cell survival, and the inhibition of JNK and p38 phosphorylation may be responsible for the inhibition of neuroinflammation.

### 3.6. SFN Treatment Significantly Inhibited NF-κB and AP-1 Signaling in LPS-Activated Microglial Cells

**Figure 6:**
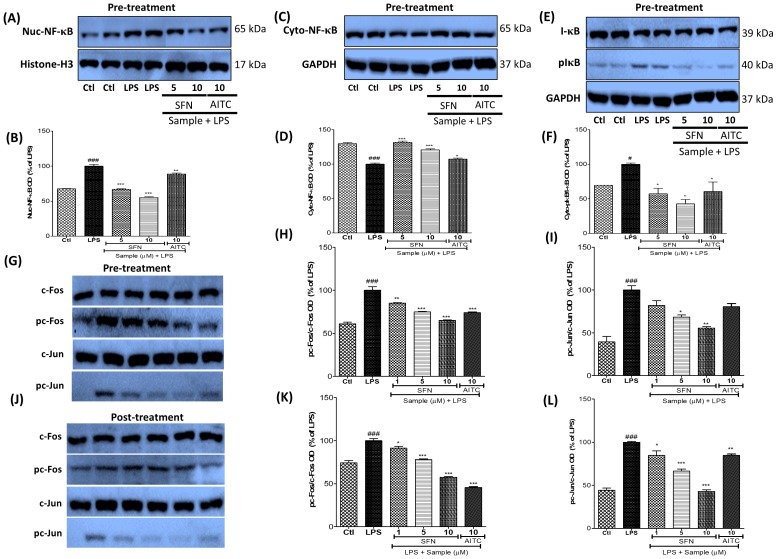
SFN inhibited NF-κB translocation and IκB phosphorylation, as well as AP-1 signaling, in LPS-activated BV2 microglial cells. The cells were pre-treated with SFN or AITC for 30 min and then stimulated with LPS (100 ng/mL). NF-κB, IκB, and pIκB expressions were observed after 1 h of LPS activation. (**A**,**B**) Nuclear NF-κB expression and band intensity; histone-H3 was used as a loading control. (**C**,**D**) Cytosolic NF-κB expression and band intensity; β-actin was used as a loading control. (**E**,**F**) Cytosolic IκB and pIκB expressions and band intensity; β-actin was used as a loading control. The LPS-stimulated cells were either pre-treated or post-treated with the indicated test chemicals. (**G**–**I**) Expression of AP-1 family proteins (pc-Jun/c-Jun and pc-Fos/c-Fos) and their band intensity after chemical pre-treatment. (**J**–**L**) Expression of pc-Jun/c-Jun and pc-Fos/c-Fos and their band intensity after chemical post-treatment. All data are presented as the mean ± standard error of the mean of three independent experiments. * *p* < 0.05, ** *p* < 0.01, and *** *p* < 0.001 indicate significant differences compared with LPS treatment alone; # *p* < 0.05 and ### *p* < 0.001 indicate significant differences compared with the untreated control group. Ctl—untreated control cells; LPS—cells treated with lipopolysaccharide only.

MAPK effector signaling controls AP-1 and NF-κB translocation and subsequently increases the transcription of inflammatory proteins. Thus, we investigated the effect of SFN on NF-κB translocation. SFN treatment significantly lowered the level of nuclear NF-κB and increased that of cytosolic NF-κB. Furthermore, it inhibited the phosphorylation of IκB. Histone-H3 was used as a loading control for the nuclear proteins, whereas β-actin was used for the cytosolic proteins. The absence of a β-actin band in the nuclear protein gel suggested the effective separation of cytosolic and nuclear proteins (Figure 6). Overall, the potency of SFN was 2-fold higher than that of AITC, as observed in the previous experiments. As MAPK signaling, especially JNK and ERK phosphorylation, directly affects AP-1 signaling, we investigated the effect of SFN on the phosphorylation of c-Fos and c-Jun, which are members of the AP-1 family. SFN significantly inhibited the phosphorylation of c-Fos and c-Jun during the pre-treatment condition, whereas it only modulated the phosphorylation of c-Jun under the post-treatment condition. Although AITC effectively inhibited the phosphorylation of AP-1, its effect was weaker than that of SFN. The fact that SFN inhibited c-Jun phosphorylation only under the post-treatment condition suggests its better usefulness for therapeutic purposes. The inhibition of JNK and ERK phosphorylation by SFN treatment might be responsible for the inhibition of c-Jun and c-Fos activation (Figure 6). 

### 3.7. SFN Treatment Significantly Inhibited the Production of Proinflammatory Cytokines in LPS-Activated Microglial Cells

**Figure 7:**
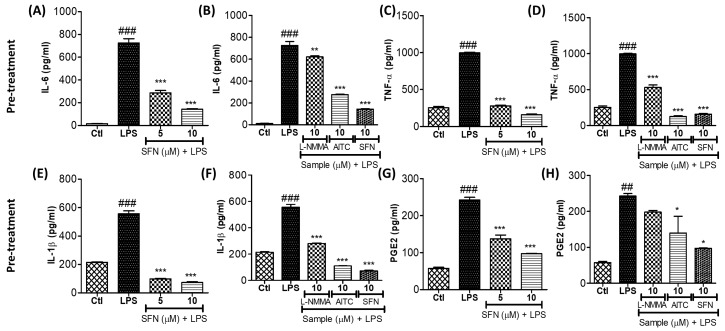
SFN inhibited proinflammatory cytokine production in LPS-activated BV2 microglial cells. The cells were pre-treated with SFN, l-NMMA, or AITC for 30 min and then stimulated with LPS (100 ng/mL). Proinflammatory cytokines in the conditioned medium of the treated cells were measured by ELISA after 24 h of LPS activation. Secretion of (**A**,**B**) IL-6, (**C**,**D**) TNF-α, (**E**,**F**) IL-1β, and (**G**,**H**) PGE2 after SFN, l-NMMA, or AITC treatment. All data are presented as the mean ± standard error of the mean of three independent experiments. * *p* < 0.05, ** *p* < 0.01, and *** *p* < 0.001 indicate significant differences compared with LPS treatment alone; ### *p* < 0.001 indicates significant differences compared with the untreated control group. Ctl—untreated control cells; LPS—cells treated with lipopolysaccharide only.

Given that SFN inhibited effector signaling and NF-κB translocation, we postulated that it must also inhibit the production of proinflammatory cytokines. The ELISAs conducted to test this hypothesis revealed that SFN treatment significantly inhibited the production of IL-6, TNF-α, IL-1β, and PGE2. This effect was almost 4-fold larger than that of l-NMMA, a well-known iNOS inhibitor. l-NMMA at 10 µM did not significantly alter PGE2 production, whereas SFN at the same concentration inhibited more than 60% of PGE2 production (Figure 7).

### 3.8. SFN Treatment Significantly Increased Anti-Inflammatory Protein (Nrf2 and HO-1) Expression and Anti-Inflammatory Cytokine (IL-10 and IL-4) Production in LPS-Activated Microglial Cells

**Figure 8:**
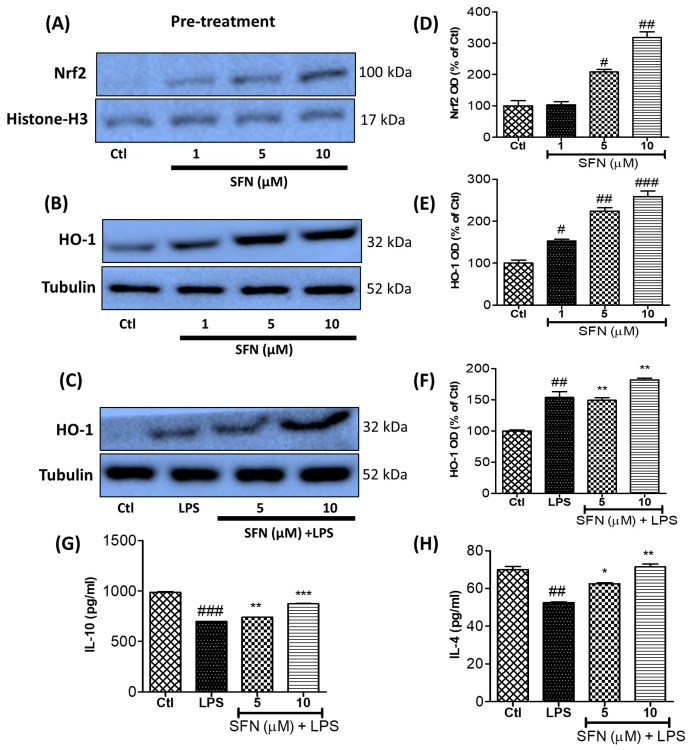
SFN increased the expression of the anti-inflammatory proteins Nrf2 and HO-1 and the anti-inflammatory cytokines IL-10 and IL-4 in BV2 microglial cells. The cells were pre-treated with SFN for 30 min and then stimulated with LPS (100 ng/mL). After 3 h and 12 h of LPS incubation, the cells were harvested and the expressions of Nrf2 and HO-1, respectively, were determined using western blot analysis. (**A**–**F**) Quantification of the Nrf2 and HO-1 expression levels. BV2 cells were pre-treated with SFN, followed by LPS (100 ng/mL) activation for 24 h. The conditioned medium was collected from the treated cells and stored for ELISA evaluation. (**G**–**H**) Secreted levels of IL-10 and IL-4, evaluated by competitive ELISA. All data are presented as the mean ± standard error of the mean of three independent experiments. * *p* < 0.05, ** *p* < 0.01, and *** *p* < 0.001 indicate significant differences compared with LPS treatment alone; # *p* < 0.05, ## *p* < 0.01, and ### *p* < 0.001 indicate significant differences compared with the untreated control group. Ctl—untreated control cells; LPS—cells treated with lipopolysaccharide only.

SFN is a well-known Nrf2 activator [19]. Nrf2 activation is followed by the increased transcriptional activation of HO-1 expression. Previous studies have also suggested that SFN-mediated anti-inflammation might occur through the activation of Nrf2 and HO-1 [20]. In the present study, we also observed that treatment with SFN alone or SFN plus LPS activation significantly upregulated the expression of the anti-inflammatory proteins Nrf2 and HO-1 in the microglia. As Lee and Chau had reported that HO-1 activation was directly related to the induced anti-inflammatory cytokine production, we also measured the secreted levels of the anti-inflammatory cytokines IL-10 and IL-4 [21]. Supporting the previous study, the same results were obtained in our study, where SFN treatment significantly increased the levels of IL-10 and IL-4 against the LPS-activated inflammation in the microglia. Altogether, these results suggest that Nrf2/HO-1 activation by SFN is capable of increasing the anti-inflammatory signaling cascade through IL-10 and IL-4 in LPS-activated microglia (Figure 8).

## 4. Discussion

In this study, we evaluated the anti-inflammatory effects of SFN pre- and post-treatments on LPS-activated BV2 microglial cells as in vitro inflammatory models of prophylactic and therapeutic treatments. We used LPS to induce the MAPK activation and subsequent inflammation of these immune cells [22,23]. SFN downregulated both LPS-induced MAPK signaling and the expression of inflammatory proteins iNOS and COX-2 under both acute and chronic microglial activation conditions. Additionally, the anti-inflammatory activity of SFN was associated with the AP-1- and NF-κB-mediated transcription of inflammatory mediators. 

As a Nrf2 activator, SFN is a well-known antioxidant with potency against oxidative stress-involving disease conditions, including inflammation and the immune response [24,25]. Previous reports have revealed that the anti-inflammatory potency of SFN was not dependent on Nrf2 activation specifically [4]. Hence, finding the key target of SFN for its anti-inflammatory effect is required. NO, a key biomarker of oxidative stress during inflammation, requires iNOS overexpression to increase its production and oxidative damage. To evaluate the antioxidative and anti-inflammatory effects of SFN, we used it to treat the microglia, which are the brain immune cells responsible for regulating the inflammatory cascades in the central nervous system. Microglial cells can be overactivated with LPS treatment, resulting in neuroinflammatory cascades; hence, the lowering of such inflammatory cascades would prove the anti-inflammatory potential of compounds against neuroinflammation. Eren et al. recently observed that SFN decreased LPS-induced inflammation, which was mediated through ERK and Nrf2 activation, and thereby exhibited potential as an antioxidant [5]. In their study, they observed that SFN treatment inhibited the production of NO, reactive oxygen species, and proinflammatory cytokines in LPS-activated N9 cells [5]. In accordance with these results, we also observed that SFN treatment inhibited NO production in the LPS-activated microglial cells. It is well known that iNOS overexpression is required for the excessive production of NO by the activated immune cells. As expected, SFN treatment significantly decreased the iNOS and COX-2 expression levels in the LPS-activated microglia. Increased COX-2 is a key element for inflammation, and its expression was also shown to be lowered by SFN treatment [26]. Qi et al. reported that SFN inhibited LPS-induced inflammation in acute lung injury in mice, where it inhibited the expression of iNOS and COX-2, and the production of PGE2 and IL-6, through the Nrf2/antioxidant response element (ARE) pathway [27]. We observed a similar result in our study, but in brain immune cells. Similarly, based on the iNOS and COX-2 expression levels, SFN showed both prophylactic and therapeutic effects in the microglia after 6 h and 24 h of LPS activation. This COX-2 inhibition may be the reason for the downregulation of PGE2 production in the activated microglia. Although the patterns of protein expression inhibition were similar, the effects of the prophylactic treatment appeared to be more potent than those of the therapeutic treatment. To inhibit these inflammatory proteins, there must be an alteration in the MAPKs; hence, we proceeded to explore these cascades.

Effector signals, such as MAPKs, are the prime cascades that are used to initiate and aggravate inflammation, degeneration, and apoptosis [28]. Although the anti-inflammatory, antioxidative, and anticancer effects of SFN have been discovered, its exact role in regulating MAPK signaling, and the respective inflammatory cascades, has not been well reported [29]. The spontaneous activation of MAPKs, the induction of inflammatory signaling, and the respective inflammatory cascades are the damage-causing factors in acute inflammatory diseases, including ischemic stroke [30]. Hence, to evaluate the role of SFN in modulating the MAPK pathways, we activated the cells with LPS for various time periods (from 30 min to 24 h) and then measured the difference in acute and chronic microglial activation resulting from SFN treatment. Under the 30 min LPS activation condition, SFN inhibited the phosphorylation of JNK, ERK, and p38 nonspecifically, where all the treated concentrations (even 1 µM) showed significant potency in inhibiting JNK phosphorylation. This also suggests that pJNK expression might be more strongly downregulated by SFN treatment. In support of this result, Jeong et al reported that a Nrf2/HO-1 activator selectively inhibited JNK phosphorylation, resulting in reduced inflammatory cascades and an increase in the anti-inflammatory cytokine IL-10 [31]. This finding indicated that HO-1 activation by SFN could be responsible for the anti-inflammatory effect—via lowered JNK and increased IL-10 secretion. Keeping this fact in mind, we further evaluated other related pathways. In our previous study, we demonstrated that AITC downregulated JNK phosphorylation and inhibited the inflammatory cascades [32]. In the present study, we observed the same pattern as well as the fact that SFN was a more potent anti-inflammatory agent than AITC was. Chronic inflammation occurs during the pathogenesis of diseases such as Alzheimer’s disease and Parkinson’s disease, where the continuous activation of inflammatory MAPK signaling, especially JNK and p38 activation, results in neuronal death and degeneration [33,34]. Hence, we examined how the chemical pre-treatment and post-treatment conditions affected LPS activation in microglia for 24 h. Under these conditions as well, the treatments of SFN and AITC both noticeably inhibited the phosphorylation of MAPK signaling proteins, with the exception of ERK signaling by SFN. A study performed by Eren et al. suggested that SFN treatment could activate the ERK signaling pathway responsible for cell survival [5]. However, the LPS-induced activation of pERK is known to be inflammatory; hence, the dual role of ERK in inflammation and cell survival has been noticed [5,35]. This might be the reason that the SFN treatment inhibited LPS-mediated ERK phosphorylation in acute microglial activation, lowering the inflammatory signal. However, ERK phosphorylation was induced after 24 h of LPS activation in the microglia, preventing cell death. Besides this, the SFN and AITC treatments inhibited the phosphorylation of JNK and p38 consistently, with the inhibition of JNK being more effective. These data suggest that SFN, as well as AITC, could be a strong candidate for the inhibition of inflammatory signaling in both acute and chronic microglial activation conditions. MAPK signaling inhibition can directly alter transcription events through the AP-1- and NF-κB-mediated pathways. The AP-1 proteins are a family of transcriptional activators, comprising c-Jun and c-Fos. Previous studies reported that SFN inhibits AP-1 in UVB-induced skin cancer, LPS-activated macrophages, and UVB-treated human keratinocytes, among others [36,37]. Supporting this result, we also observed the same effect in LPS-activated microglia treated with SFN. The activation of c-Jun and c-Fos can induce transcriptional proteins in an immune cell-mediated inflammatory condition. JNK activation can rapidly phosphorylate c-Jun, but its role in c-Fos phosphorylation is not known [38]. Another independent study revealed that ERK phosphorylation is responsible for the regulation of c-Fos transcriptional activity [39]. In our study, SFN treatment inhibited the phosphorylation of c-Jun and c-Fos. The inhibition of c-Jun phosphorylation may be due to the lowered JNK phosphorylation, whereas that of c-Fos phosphorylation may be because of the reduced ERK phosphorylation. We therefore suggest that SFN is a single AP-1 inhibitor that lowers the activation of both c-Jun and c-Fos through JNK and ERK inhibition, respectively. SFN treatment may also inhibit NF-κB translocation, IκB degradation, and IκB phosphorylation. This suggests that SFN inhibits not only MAPK signaling but also the NF-κB- and AP-1-mediated transcription of inflammatory proteins. Although our study supports the previous findings of the anti-inflammatory effects of SFN, it also shows that the control of this phytochemical over effector signaling and transcriptional regulation is through downregulation of the JNK, NF-κB, and AP-1 cascades. SFN can further lower the transcription of inflammatory protein mediators and proinflammatory cytokines, in particular, resulting in the inhibition of inflammatory cascades and a low risk of neurodegeneration and toxicity. Proinflammatory cytokines are the key toxic mediators responsible for neurodegeneration and neuronal death, resulting in conditions such as Alzheimer’s disease and Parkinson’s disease. The SFN-mediated inhibition of MAPK, AP-1, and NF-κB may lower the production of those cytokines. As we expected, both the SFN and AITC treatments inhibited the production of IL-6, IL-1β, and TNF-α in the LPS-activated microglia. Previous studies revealed that JNK signaling specifically controls the transcription of inflammatory signals, such as iNOS, NO, IL-1β, and TNF-α [40,41]. One study revealed that JNK is a key signal responsible for the increase in TNF-α and IL-1β production and the subsequent neurotoxicity [41,42]. SFN dramatically lowered JNK phosphorylation and subsequently c-Jun and c-Fos phosphorylation, as well as NF-κB-mediated transcription. The high potency of the SFN-mediated inhibition of TNF-α and IL-1β was also observed. Therefore, this suggests that JNK inhibition must be a key effect in this cascade and that it was controlled by SFN. 

Broccoli sprout extract and SFN itself are known as Nrf2/HO-1 activators [43]. In this study, we further confirmed that SFN treatment significantly increases Nrf2 and HO-1 activation in microglial cells. In addition to this, we evaluated the production of IL-10 and IL-4 in LPS-activated microglia treated with SFN, where the levels of these anti-inflammatory cytokines were markedly increased compared with their levels in cells treated with LPS only. The inhibition of NF-κB- and AP-1-mediated transcription might be responsible for the downregulated transcription of inflammatory cytokines, whereas activation of Nrf2-mediated transcription was responsible for the upregulation of the anti-inflammatory HO-1, IL-4, and IL-10 proteins. Collectively, these effects resulted in the lowered inflammation and increased anti-inflammatory events. Altogether, our results suggest that the SFN-mediated Nrf2/HO-1 activation regulated by JNK inhibition is responsible for the increase in IL-10 production and the downregulation of inflammatory cytokines against LPS-activated microglia. These events might be responsible for the anti-inflammatory and neuroprotective effects of SFN.

## 5. Conclusions

**Figure 9:**
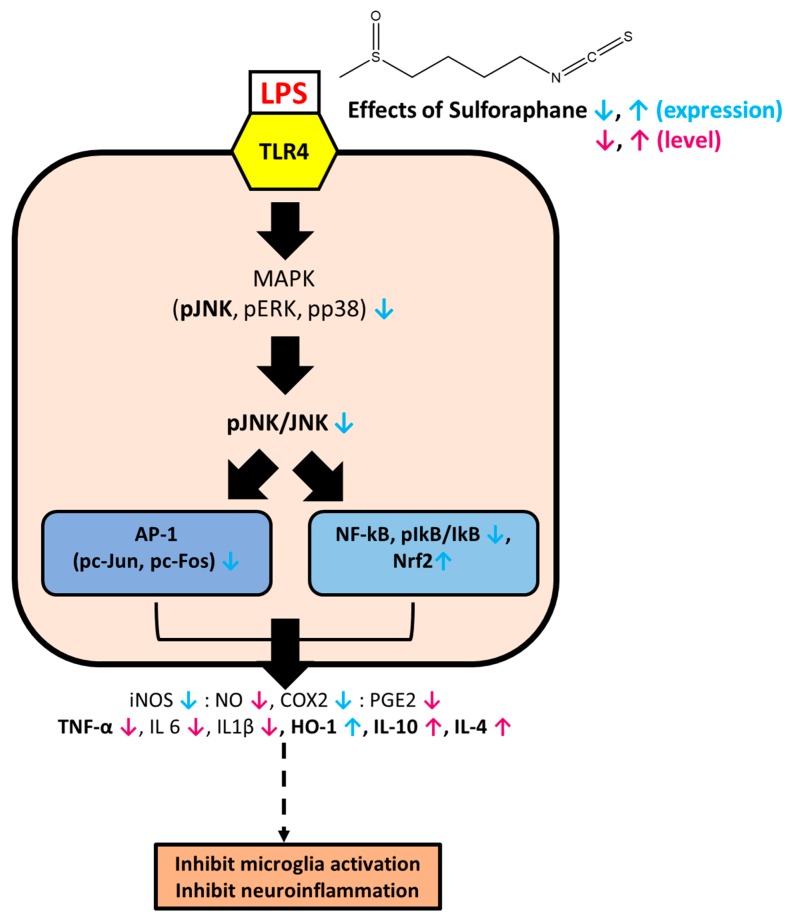
Hypothetical model of the SFN-mediated anti-inflammatory effect via JNK/AP-1/NF-κB pathway inhibition and Nrf2/HO-1 pathway activation, with subsequent upregulation of IL-10 and IL-4.

A well-known Nrf2/HO-1 activator, SFN, not only amplified the level of the anti-inflammatory cytokines IL-4 and IL-10 but also significantly downregulated pJNK/JNK-mediated inflammatory signaling, which was followed by the reduced NF-κB-/AP-1-facilitated transcription of inflammatory mediators, such as iNOS, COX-2, NO, and PGE2, and proinflammatory cytokines (Figure 9). Therefore, this study suggests that SFN could be a potential prophylactic or therapeutic agent for the inhibition of microglial cell activation and the subsequent inflammatory cascades against brain immune cells. If these findings can be confirmed in vivo and in clinical trials, SFN would be an attractive candidate for the regulation of the inflammatory responses in brain diseases where neuroinflammation is a central hallmark. Further studies to observe SFN-mediated JNK knockdown in vivo are being planned. 

## Supplementary Materials

Supplementary materials are available online at https://www.mdpi.com/2073-4409/8/2/194/s1.
