# Supplementary material for: Anti-Inflammatory Effect of Sulforaphane on LPS-Activated Microglia Potentially through JNK/AP-1/NF-κB Inhibition and Nrf2/HO-1 Activation
Source: Cells. 2019 Feb 22;8(2):194. doi: 10.3390/cells8020194 (PMC6406309; doi:10.3390/cells8020194)

cells

MDPI

Article

Anti-Inflammatory Effect of Sulforaphane on LPS-Activated Microglia Potentially through JNK/AP-1/NF-κB Inhibition and Nrf2/HO-1 Activation

Lalita Subedi [1], Jae Hyuk Lee [1], Silvia Yumnam [1], Eunhee Ji [2] and Sun Yeou Kim [1,3,4] *

[1] Laboratory of Pharmacognosy, College of Pharmacy, Gachon University, #191, Hambakmoero, Yeonsu-gu, Incheon 21936, Republic of Korea; subedilali@gmail.com (L.S.); wogur6378@naver.com (J.H.L.); silviayumnam@gmail.com (S.Y.)

[2] Laboratory of Clinical Pharmacy, College of Pharmacy, Gachon University, #191, Hambakmoero, Yeonsu-gu, Incheon 21936, Republic of Korea; ehji@gachon.ac.kr

[3] Gachon Institute of Pharmaceutical Science, Gachon University, 191, Hambakmoe-ro, Yeonsu-gu, Incheon 21936, Korea

[4] Gachon Medical Research Institute, Gil Medical Center, Incheon 21565, Korea

* Correspondence to: sunnykim@gachon.ac.kr; Tel.: +82-32-820-4931; Fax: +82-32-820-4932

Received: date; Accepted: date; Published: date

Supplementary Figure S1.

RAW 264.7 and THP-1cells were pre-treated with SFN 30 min before LPS activation and incubated for 24 h to check the production of nitrite and cell viability, 6 h for the expression of iNOS/COX-2 and 30 min LPS activation for the evaluation of MAPK signaling proteins. (A-B) Nitrite production and cell viability in RAW 264.7 cell. (C-E) expression of iNOS/COX-2 and MAPK proteins in RAW 264.7 cell. (F-G) Nitrite production and cell viability in THP-1 cell. (C-E) expression of iNOS/COX-2 and MAPK proteins in THP-1 cell. All data are presented as the mean ± standard error of the mean of three independent experiments. * $p < 0.05$, ** $p < 0.01$, and *** $p < 0.001$ indicate significant differences compared with treatment with LPS alone, while # $p < 0.05$, ### $p < 0.001$ indicates significant differences compared with an untreated control group.

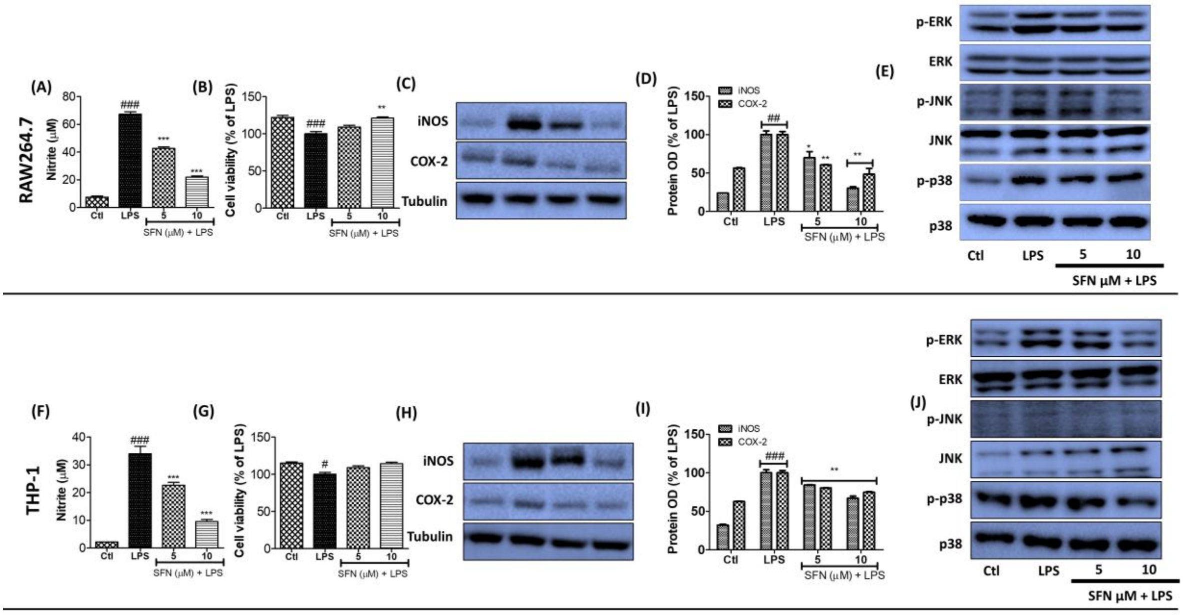

Supplement: Supplementary file 1 [file cells-08-00194-s001.pdf]
